# Dr. Balfour, on Rheumatism

**Published:** 1817-04

**Authors:** William Balfour

**Affiliations:** Edinburgh


*To the Editors of the Medico-Chirurgical Journal & Review.*

GENTLEMEN,

IN Number XIV. of your Journal there is a paper on the Oriental Mode of curing Chronic Rheumatism, in which the author gratuitously assumes that my mode of treating the disorder is the same with that practised, "time immemorial, in the Eastern world;" and hence concludes, that my "mode of cure is not new, even in this country."

Before drawing any such conclusion, however, it was incumbent on the author of the paper in question to shew, that the practice of the East is conducted on the same principles, in the same manner, and with the same effects, with mine. His telling a thirty-year-old story is certainly not sufficient to invalidate my claim to discovery. That champoeing is practised in India, both as a matter of luxury and for rheumatism, and that stuffed or wooden hammers are also used, especially in China, I do not dispute. Of these facts I was informed, for the first time, in June, 1815, by a gentleman whom I accidentally met at Perth, and who had repeatedly visited China. My Treatise on Rheumatism, however, was *published* in the Edinburgh Medical and Surgical Journal in *April* 1815. It was impossible, therefore, that I could have been indebted, in the slightest degree, to the practice of the East. My principles and practice in rheumatism are the pure offspring of my own observation and experience.

But it is no uncommon thing for men in different countries to make the same discoveries, without the smallest communication with one another on the object of their pursuits. Thus, Scheele in Sweden, Priestley in Britain, and Lavoisier in France, discovered oxygen gas much about the same time. Each of these philosophers, therefore, is entitled to the whole merit of the discovery. Let us suppose, that oxygen gas was known to the Chinese 2000 years ago, but that we still remained ignorant of it in this country; would a philosopher, who should make the same discovery in our day, have less merit, because it was known in another country ages before he was born? Farther, important discoveries have been made, neglected, and forgotten, for a great length of time, till some fortunate inquirer has made them anew. When any thing of this kind occurs, a certain class of men are stimulated, by malice and envy, to search the records of past times; and thus, with a view of injuring the living, justice is ultimately done to the dead. This actually took place with regard to the discovery of oxygen gas. Mayow discovered the compound nature of the atmosphere, considerably more than 100 years before Scheele, Priestley, and Lavoisier; and attributed to something in it all the qualities that are now ascribed to oxygen gas: he was, therefore, the first discoverer of this gas. It will not from hence be contended, however, that the discovery of Scheele, of Priestley, and of Lavoisier, is the less brilliant, or the less real, since they were altogether unacquainted with the works of Mayow. In the same way, my friend, the justly celebrated Dr. William Wright, of Edinburgh, discovered the utility of the cold affusion in locked-jaw (of which he has given an account in the London Medical Observations and Inquiries, vol. vi.), ignorant at the time that Celsus recommends the same remedy.

I do not rest my defence here. The practice of the East is conducted on no principle, is essentially different from mine in its mode of application, and is not in the thousandth degree so efficient. Had Dr. Lind, of Haslar, seen the effects of but one of my operations in a recent case of rheumatism, he would have adopted my practice and continued it, not one year only, but all the years of his life. Any person that chuses to make himself acquainted with the principles upon which I conduct the cure of rheumatism, will soon be satisfied of the inferiority of the practice of the East. Indeed, I have the unexceptionable testimony to this effect, of officers who have served in India, and who are experimentally acquainted with both modes of cure. The gentleman whose paper has called forth these observations, must never have read my Treatise on Rheumatism; otherwise, he would have seen that my practice is equally efficient in the *acute* as in the *chronic* form of the disease. This he does not pretend to be the case with the Eastern practice; which, therefore, instead of being an improvement on mine, comes infinitely short of it.

In my principles and practice in the cure of rheumatism, I have gone farther beyond the Eastern practice than Harvey, when he discovered the circulation of the blood, went beyond his master Fabricius. Fabricius demonstrated valves in the veins that would permit a fluid to pass to the heart, but not to return. The easy conclusion was, if a fluid passes to the heart, it must come from the heart; and hence a circuit is performed. Champoeing and the hammer can affect the body superficially only, and their application must be temporary; my mode of applying percussion affects the whole body, internally as well as externally; and my mode of compression can be regulated at pleasure.

In introducing compression and percussion for the cure of rheumatism, I have as good a claim to discovery as Dr. Jenner, in introducing vaccination. The fact, that cow-pox was a preventive of small-pox, must have been known for centuries to hundreds of people. It was reserved for Dr. Jenner to produce the disease artificially, and thus render the blessing universal. I had no such light to guide me, either from the East, or from the West; from the North, or from the South.

It is very surprising, that a physician of Dr. Lind's sagacity and skill, should have adopted a practice imported from a foreign country; and finding it efficient, should drop it in a year! It is the first instance on record, I believe, of any practice having ever been dropped that was found successful in an obstinate disease. It was asserted, when I first published my discovery with regard to the utility of bandages in the cure of rheumatism, that they had been employed long before, in the Royal Infirmary of Edinburgh, for the same purpose; but I never yet could find one who saw them so employed. I inquired of all the Professors, if they ever prescribed them in their practice? When they declared they never did; nor ever heard of bandages being used, *qua* bandages, in rheumatism.

It will be conceded, I presume, by most men of candour, that had a practice existed in the East, equally efficient as my practice in rheumatism will be found, it could not have escaped the observation of British practitioners, or have remained unknown to Professors of a Medical School so celebrated as that of Edinburgh, and to which there is every season an afflux of students from every quarter of the world. None, therefore, will deny me the honour of having discovered the utility of compression and percussion in the cure of rheumatism, but those who would ascribe a discovery to any nation, or to any age, rather than to a countryman and contemporary.

WILLIAM BALFOUR.

Edinburgh, March 10, 1817.

---

*As the paper referred to in the above letter was not inserted with any view to detract in the slightest degree from the just merits of Dr. B. so have the Editors now offered him an opportunity of vindicating his claim to what he considers an important discovery. The public must ultimately decide on the utility of the practice, and duly apportion the honour and priority of invention to the various claimants.*

